# Glycine Ameliorates Endoplasmic Reticulum Stress Induced by Thapsigargin in Porcine Oocytes

**DOI:** 10.3389/fcell.2021.733860

**Published:** 2021-11-30

**Authors:** Sicong Yu, Lepeng Gao, Chang Zhang, Yumeng Wang, Hainan Lan, Qianran Chu, Suo Li, Xin Zheng

**Affiliations:** College of Animal Science and Technology, Jilin Agricultural University, Changchun, China

**Keywords:** endoplasmic reticulum stress, glycine, apoptosis, oocyte meiotic maturation, embryo development (*in vitro*)

## Abstract

The endoplasmic reticulum (ER) is a multifunctional organelle in the cytoplasm that plays important roles in female mammalian reproduction. The endoplasmic reticulum and mitochondria interact to maintain the normal function of cells by maintaining intracellular calcium homeostasis. As proven by previous research, glycine (Gly) can regulate the intracellular free calcium concentration ([Ca^2+^]_i_) and enhance mitochondrial function to improve oocyte maturation *in vitro*. The effect of Gly on ER function during oocyte *in vitro* maturation (IVM) is not clear. In this study, we induced an ER stress model with thapsigargin (TG) to explore whether Gly can reverse the ER stress induced by TG treatment and whether it is associated with calcium regulation. The results showed that the addition of Gly could improve the decrease in the average cumulus diameter, the first polar body excretion rate caused by TG-induced ER stress, the cleavage rate and the blastocyst rate. Gly supplementation could reduce the ER stress induced by TG by significantly improving the ER levels and significantly downregulating the expression of genes related to ER stress (Xbp1, ATF4, and ATF6). Moreover, Gly also significantly alleviated the increase in reactive oxygen species (ROS) levels and the decrease in mitochondrial membrane potential (ΔΨ m) to improve mitochondrial function in porcine oocytes exposed to TG. Furthermore, Gly reduced the [Ca^2+^]_i_ and mitochondrial Ca^2+^ ([Ca^2+^]_m_) levels and restored the ER Ca^2+^ ([Ca^2+^]_ER_) levels in TG-exposed porcine oocytes. Moreover, we found that the increase in [Ca^2+^]_i_ may be caused by changes in the distribution and expression of inositol 1,4,5-triphosphate receptor (IP_3_R1) and voltage-dependent anion channel 1 (VDAC1), while Gly can restore the distribution and expression of IP_3_R1 and VDAC1 to normal levels. Apoptosis-related indexes (Caspase 3 activity and Annexin-V) and gene expression Bax, Cyto C, and Caspase 3) were significantly increased in the TG group, but they could be restored by adding Gly. Our results suggest that Gly can ameliorate ER stress and apoptosis in TG-exposed porcine oocytes and can further enhance the developmental potential of porcine oocytes *in vitro*.

## Introduction

The endoplasmic reticulum (ER) is a multifunctional organelle in the cytoplasm that plays important roles in female mammalian reproduction. Several studies have demonstrated that enhanced ER function can improve porcine, mouse, and cattle oocyte maturation and parthenogenetic embryonic development *in vitro* (Zhang et al., 2012a; Zhang et al., 2012b; Sutton-McDowall et al., 2016). These functions are related to the regulation of calcium (Ca^2+^) by the ER.

The ER is the key system for Ca^2+^ storage and release, and mitochondria are the major effectors of Ca^2+^ absorption (Chevet et al., 2001; Groenendyk and Michalak, 2005; Larner et al., 2006). The ER and mitochondria are connected by mitochondria-associated membranes (MAMs) and interact to maintain the normal function of cells by maintaining intracellular calcium homeostasis (Ando et al., 2018). An imbalance in calcium homeostasis can activate ER stress, which can lead to the transfer of ER Ca^2+^ ([Ca^2+^]_ER_) to the cytoplasm and can cause an increase in the intracellular free calcium concentration ([Ca^2+^]_i_). When there is an overload of Ca^2+^ in oocyte mitochondria ([Ca^2+^]_m_), it is accompanied by an increase in reactive oxygen species (ROS) and damage to metabolic homeostasis, which can then cause damage to mitochondrial function, block the meiotic process of oocytes and reduce the developmental ability of oocytes (Zhao et al., 2016).

Normally, intracellular Ca^2+^ homeostasis is dependent mainly on ryanodine receptors (RyRs), inositol-1,4,5-trisphosphate receptors (IP_3_Rs) and sarco-endoplasmic reticulum Ca^2+^ ATPase pumps (SERCA pumps) in the ER (Santulli et al., 2017). RyRs and IP_3_Rs release Ca^2+^ from the ER lumen into the cytoplasm or mitochondria, and SERCA takes up free cytosolic Ca^2+^ into ER stores (Bootman et al., 2001). In the IP_3_R channel, Ca^2+^ stored in the ER is released through IP_3_R, diffuses through the MAMs, is taken up by voltage-dependent anion channel 1 (VDAC1), and is transported to the mitochondrial matrix by the mitochondrial Ca^2+^ uniporter (MCU). This process maintains the dynamic Ca^2+^ equilibrium. An imbalance in intracellular calcium homeostasis can induce ER dysfunction and ER stress. The inhibition of SERCA activity by thapsigargin (TG) caused an imbalance in Ca^2+^ homeostasis and induced the occurrence of ER stress (Wu et al., 2012). Excessive Ca^2+^ influx into mitochondria causes the opening of mitochondrial permeability transition pores, resulting in the production of ROS, and leads to the release of proapoptotic proteins, such as cytochrome C (Cyto C), which can not only influence the developmental potential of oocytes but can also induce cell apoptosis (X. M. Zhao et al., 2016).

ER stress could activate the unfolded protein response (UPR), which is a protective pathway that maintains intracellular homeostasis. The major UPR-associated pathways include the PERK, IRE1, and ATF6 pathways, which involve downstream molecules such as CHOP and Caspase 3/9/12 (Siman et al., 2001; Hitomi et al., 2004), Xbp1 and Bip/Grp78, and ATF4 (Mei et al., 2013; Fung and Liu, 2014; Jheng et al., 2014). In pigs, negative effects on oocyte maturation and embryo development occur as a result of ER protein misfolding and apoptosis (Lin et al., 2016). However, research shown that the oocyte maturation rate and embryonic development can be recovered or even improved when ER stress is reduced (Ridlo et al., 2021). Inhibition of the ER stress-related PERK pathway could significantly improve the embryonic development of aged mouse oocytes (Takehara et al., 2020). In addition, it was previously reported that suppression of ER stress could significantly increase the maturation rate and decrease ROS levels and apoptosis in bovine oocytes (Khatun et al., 2020). Therefore, we may improve the maturation rate of porcine oocytes by inhibiting ER stress in the *in vitro* maturation (IVM).

Glycine (Gly) is an important component of the antioxidant glutathione (GSH) and is involved in a variety of intracellular signalling pathways in mammals. Gly is ubiquitously distributed in oviduct fluid, uterine fluid and follicular fluid in sows and plays an important role in cell protection mechanisms against oxidative damage from ROS. Several studies have shown that Gly plays a crucial role in the development of oocytes (Zander-Fox et al., 2013). In fact, we conducted basic research on the effect of Gly on porcine oocyte IVM in the early stage (Li et al., 2018; Wang Y. et al., 2020). Our previous studies showed that 6 mM Gly treatment has a beneficial effect on oocyte IVM and subsequent blastocyst development after parthenogenetic activation (PA) by decreasing ROS levels and increasing mitochondrial function (mitochondrial ΔΨ m, ATP concentration) to reduce apoptosis and regulate gene expression related to development (FGFR2 and Hsfl) and apoptosis (Bax and Bcl2). Furthermore, we also proved that Gly could improve oocyte IVM by regulating the [Ca^2+^]_i_ to enhance mitochondrial function (Yu et al., 2021).

But the effect of Gly on ER stress during oocyte IVM is still unclear. Based on the above research, the objective of this study was to confirm the role of Gly in ameliorating ER stress induced by TG and whether Gly is related to calcium regulation in porcine oocytes.

## Materials and Methods

### Animals and Ethics

All experiments were performed at Jilin Agricultural University, China. Ethical approval for the present study was obtained from the Ethics Committee of Jilin Agricultural University.

### Oocyte Collection and IVM of Oocytes

The porcine ovaries were obtained from a local slaughterhouse and sent to the laboratory within 2 h at 25–30°C in 0.9% saline (w/v). Cumulus-oocyte complexes (COCs) were drawn from follicles with a diameter of 3–6 mm with an 18-gauge needle attached to a 20-ml disposable syringe. Before COCs were cultured in a four-well dish that contained 500 μl of IVM medium (NCSU-37 supplemented with 10 IU/ml pregnant mare serum gonadotropin (PMSG), 10 IU/ml human chorionic gonadotrophin (hCG) and 10 μg/ml epidermal growth factor (EGF)) in each well, they were washed 3 times with TL-HEPES-PVA (polyvinyl alcohol, 0.1%). They were incubated at 38.5°C under 5% CO_2_ in 95% humidified air for IVM. After 22 h of culture, the previous medium was replaced with the same medium without PMSG/hCG, and then the culture was continued for 22 h.

First, the control group and the treatment group supplemented with 50 nM TG were cultured. After 22 h of culture, the media in the control group and one of the TG treatment groups were replaced with the new control medium to eliminate TG, and the media in the remaining TG treatment groups were replaced with 6 mM Gly medium (Control group, TG group and TG + 6 mM Gly group). PMSG/hCG were no longer added to the new medium.

### Measurement of the Degree of Cumulus Cell Expansion

The diameter expansion of cumulus cells was measured by microscopy (ix70, Olympus, Tokyo, Japan). COCs were measured using the two vertical diameters at maturity in all groups, and a total of 30 were measured in each group. In addition, the cumulus cells were denuded by 0.1% hyaluronidase. The marker of nuclear maturation is the discharge of the first polar body (PB1), and mature oocytes were collected for subsequent trials (Supplementary Figure S1).

### Parthenogenetic Activation of Oocytes

The collected oocytes were activated with a direct current pulse (1.2 kV/cm, 30 μs) utilizing a BXT Electro-Cell Manipulator 2001 (BXT Inc., San Diego, CA) in 0.28 mol/L mannitol containing 0.1 mM MgSO_4_ and 0.05 mM CaCl_2_. After PA, oocytes were cultured in NCSU-37 medium [4 mg/ml bovine serum albumin (BSA) and cytochalasin B (CB)] for 3 h and then transferred into *in vitro* culture medium in the same culture environment as IVM for 2 days to record their morphology and cleavage rate. The blastocyst formation rate was recorded on day 7.

### Measurement of Intracellular GSH and ROS Levels in Oocytes

The intracellular GSH and ROS levels of oocytes at the metaphase II (MII) stage were measured by CellTracker Blue CMF2HC (4-chloromethy-6.8-difluoro-7-hydroxycoumarin; Invitrogen) and H_2_DCFDA (2′,7′-dichlorodihydrofluorescein diacetate) fluorescence assays. Fluorescence staining was performed as described in a previous report (Yu et al., 2021). The fluorescence was observed under a fluorescence microscope (Olympus Tokyo, Japan) and imaged with ImageJ software.

### Determination of the Mitochondrial Membrane Potential in Oocytes

The mitochondrial ΔΨ m was analysed by a mitochondrial membrane potential assay kit with JC-1 according to the manufacturer’s protocol to determine the change in ΔΨ m in oocytes. After washing, mature-denuded oocytes were washed three times with PBS–PVA and stained for 15 min with fluorescent dyes at 38.5°C. Then, the oocytes were washed three times, examined with a Nikon fluorescence microscope (Tokyo, Japan) and analysed by ImageJ software.

### Measurement of [Ca^2+^]_ER_, [Ca^2+^]_m_, and [Ca^2+^]_i_ Levels

The [Ca^2+^]_i_ levels were detected as described in a previous report (Yu et al., 2021). Briefly, oocytes were stained with 5 μM Fluo-3/AM (Beyotime) for 40 min at 38.5°C and then washed three times with PBS-PVA (without Ca^2+^). The [Ca^2+^]_ER_ levels were assessed by Mag-Fluo-4 AM, and the oocytes were incubated with 10 μM Mag-4 AM for 15 min at 37°C. The cells were washed three times with PBS-PVA and incubated without Mag-Fluo-4 AM. The [Ca^2+^]_m_ levels were stained with 10 μM Rhod-2 AM, and the dyeing operation was the same as that for Mag-Flou-4 AM. Finally, the fluorescence intensity was detected with an Olympus fluorescence microscope (Tokyo, Japan) and analysed by ImageJ software. The final average fluorescence was calculated from the fluorescence intensity of all oocytes.

### Staining of Endoplasmic Reticulum in Oocytes

ER richness were evaluated using the ER-Tracker Red (Molecular Probes). And fluorescently labelled ER-Tracker Red was used to quantify the ER mass. Mature-denuded oocytes were washed in PBS-PVA three times and stained with ER-Tracker Red (1:200) for 20 min at 37°C. Images of oocytes were captured using an Olympus fluorescence microscope (Tokyo, Japan) after washing three times. ImageJ software was used to quantify ER mass. The fluorescence signal was calculated as the average intensity after background subtraction.

### Measurement of Caspase 3 Activity and Apoptosis

The Caspase 3 Activity and Apoptosis Detection Kit for Live Cells (Beyotime) was used to determine Caspase 3 activity and Annexin-V signals in oocytes following the instructions. The oocytes were directly added to the solution prepared using the kit and incubated for 20 min for fluorescence microscopy detection (Olympus, Tokyo, Japan). Then, the oocytes were viewed with a fluorescence microscope, and the fluorescence intensities of oocytes were assayed by the ImageJ program.

### Immunofluorescence

MII oocytes subjected to different treatments were immobilized with 4% paraformaldehyde for 30 min at room temperature (RT) and then washed three times in PBS-PVA for 5 min each time. Next, MII oocytes were permeabilized with 1% Triton X-100 at RT for at least 10 min and transferred to blocking solution (PBS +3% BSA) for 1 h. For IP_3_R1 or VDAC1 staining, oocytes were incubated overnight at 4°C with primary antibodies [rabbit polyclonal anti-IP_3_R1 (Invitrogen, PA1901), 1:500; VDAC1 rabbit monoclonal antibody (Beyotime, AF1027), 1:200]. After three washes in PBS-PVA for 10 min each time, oocytes were incubated with goat antirabbit IgG [(Invitrogen A27034), 1:250] for 1 h at RT in the dark. Then, oocytes were stained with 10 μg/ml Hoechst 33,342 at 37°C for 10 min. Finally, samples were mounted on glass slides. Distribution patterns of IP_3_R1 and VDAC1 were identified by laser-scanning confocal microscopy (Leica TCS SP5). Twenty oocytes were assayed in each group.

### Quantitative Real-Time PCR

For analysis of gene expression, 50 mature denuded oocytes in each group were placed in 1.5-ml centrifuge tubes and stored at −80°C until RNA was extracted utilizing the Arcturus^®^ PicoPure^®^ RNA Isolation Kit (Thermo Fisher Scientific, KIT0204, United States) according to the manufacturer’s instructions. The concentration of extracted RNA was measured employing a NanoDrop 2000c Spectrophotometer (Thermo Fisher Scientific). The PrimeScript™ RT reagent Kit with gDNA Eraser (Perfect Real Time) was utilized to synthesize complementary DNA (cDNA). The CFX96 Real-Time PCR Detection System was utilized for real-time PCR (Yu et al., 2021). Specific primer sequences (Table 1) were devised utilizing the NCBI database. Relative gene expression levels were calculated utilizing the 2^−∆∆CT^ strategy, and 18S ribosomal RNA (18S) served as the loading control. For helpful comparison, the average expression level of each gene was normalized to the control group.

**TABLE 1:** Primer sequences for RT-PCR.

Gene	Primer sequences (5′-3′)	Product length (bp)
Bax	F: CCGAAATGTTTGCTGACG	232
	R: AGC​CGA​TCT​CGA​AGG​AAG​T	
Bcl2	F: ACC​TGA​ATG​ACC​ACC​TAG​AGC	281
	R: TCCGACTGAAGAGCGAAC	
Caspase 3	F: TTG​GAC​TGT​GGG​ATT​GAG​ACG	165
	R: CGC​TGC​ACA​AAG​TGA​CTG​GA	
Cyto C	F: CTGCGAGTGGTGGATTGT	733
	R: ATG​CCT​TTG​TTC​TTG​TTG​G	
18S	F: ACGTTGGCGAGAGCGTG	82
	R: AGGTGGAGGAGGCGAGAG	
Grp78	F: ATA​TAA​GCG​GAG​CAG​GCG​AC	87
	R: GAG​CTC​TCA​CAC​ACA​CGG​AA	
ATF4	F: AGT​CCT​TTT​CTG​CGA​GTG​GG	80
	R: CTG​CTG​CCT​CTA​ATA​CGC​CA	
ATF6	F: GGG​AAA​GAT​TCC​ACT​TGG​TCT​TA	172
	R: TCT​CCC​AAG​GCA​TCA​AAT​CCA​A	
Xbp1	F: AGG​GAA​AGA​TTC​CAC​TTG​GTC​TTA	182
	R: CAA​ATG​CCT​GTC​TCC​CAA​GG	
IP_3_R1	F: CAG​ATG​GTG​GAC​AAC​TCA​GGC	246
	R: GGC​TTT​GGA​ACT​CGT​GGC​AGA	
CALR	F: TGG​GAC​AAG​CCT​GAA​CAC​AT	102
	R: TGG​GTT​CTG​TAT​CAC​TGG​CG	
VDAC1	F: AGA​AGA​TGG​CTG​TGC​CTC​CT	167
	R: CAC​TTT​GGT​GGT​CTC​CGT​GT	
Grp75	F: AAG​TAG​GGC​AGG​GGA​AGC​TA	120
	R: AGG​CCA​CAG​GGT​AGC​TAG​AA	

### Statistical Analysis

All experiments were repeated at least three times. The data are expressed as the mean ± standard error of the mean (SEM). Statistical analysis utilized univariate analysis of variance (ANOVA) followed by Duncan’s multiple range test conducted with the IBM-SPSS 23.0 statistical program. To ensure the homogeneity of variance, the percentage data were arcsine-transformed before analysis. Differences in gene expression were compared by Student’s *t*-test, and *p* < 0.05 was considered statistically significant.

## Results

### Effect of Gly on the Cumulus Expansion and Maturation Efficiency of Porcine Oocytes Treated With TG

We first detected the effects of Gly on cumulus expansion and the oocyte maturation rate. The results showed that Gly is not only conducive to cumulus diffusion but also improves the IVM rate of porcine oocytes (Supplementary Figure S2).

**FIGURE 1:**
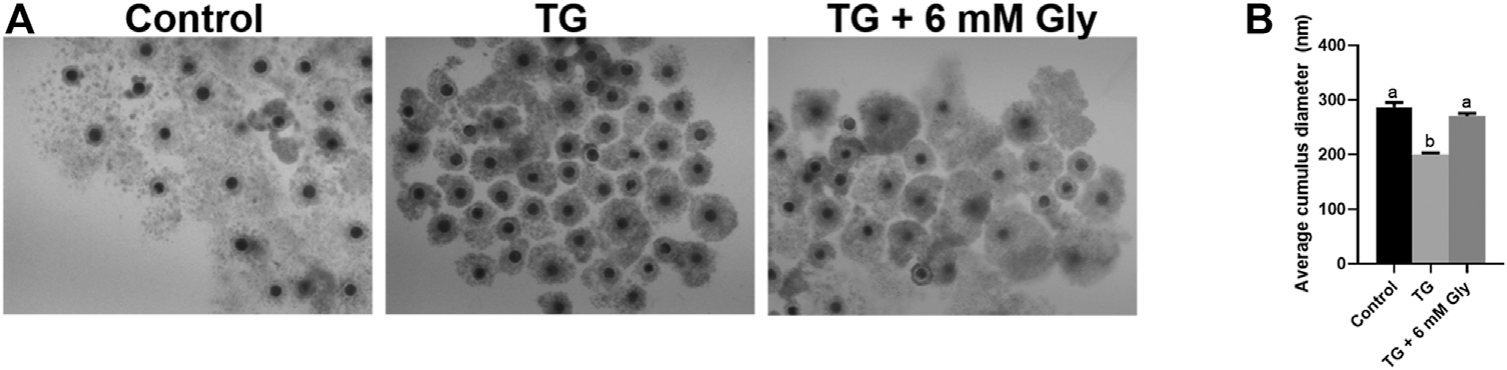
Effect of Gly on the cumulus expansion of porcine oocytes treated with TG. Measurement of the cumulus cell expansion in COCs after TG treatment or Gly supplementation. **(A)** The morphology of COCs matured *in vitro*. **(B)** The diameters of cumulus cell expansion were measured in all groups. *n* = 3 experimental replicates with more than 30 COCs per treatment group. Bar = 200 µm. Different superscript letters denote a significant difference (^a, b^ *p* < 0.05).

Then we detected the effects of Gly on cumulus expansion and the oocyte maturation rate after exposure to TG. The detection indexes were the expansion diameter of cumulus cells and the PB1 extrusion rate. The results showed that both cumulus expansion and the oocyte maturation rate were decreased by TG. The diameter of COCs was measured and recorded after IVM (Figures 1A,B). As expected, the cumulus expansion diameter was significantly reduced in the TG-treated group (*p* < 0.05). Importantly, the cumulus expansion of oocytes exposed to TG returned to the control level after Gly treatment.

The oocyte maturation rate and death rate showed similar results. Compared with the rates of the control group and TG + 6 mM Gly group (73.05 ± 3.01, 67.30 ± 1.98), the maturation rate was decreased significantly in the TG treatment group (54.63 ± 2.2, *p* < 0.05). There were no significant differences between the control group and the TG + 6 mM Gly group. The death rate of the TG group was significantly higher than the death rates of the control group and the TG + 6 mM Gly group, but the rescue group was not significantly different from the control group (Table 2).

**TABLE 2:** Effect of Gly on the *in vitro* maturation of porcine oocytes treated with TG.

Group	Oocytes examined	Oocytes (%)		
		Immature %	Degeneration %	MII %
Control	558	16.17 ± 3.26	12.17 ± 2.89^b^	73.05 ± 3.01^a^
TG	345	24.14 ± 6.62	24.58 ± 4.11^a^	54.63 ± 2.21 ^b^
TG + 6 mM Gly	306	20.35 ± 4.57	14.14 ± 3.39^ab^	67.30 ± 1.98^a^

The number of oocytes examined (1209) is the total of four independent assays. ^a,b^ Different superscripts within the same column differ significantly (*p* < 0.05). MII, metaphase II.

Our results indicated that Gly could effectively restore the cumulus expansion and maturation of porcine oocytes that were exposed to TG.

### Effect of Gly on GSH and ROS Levels in Porcine Oocytes Treated With TG

**FIGURE 2:**
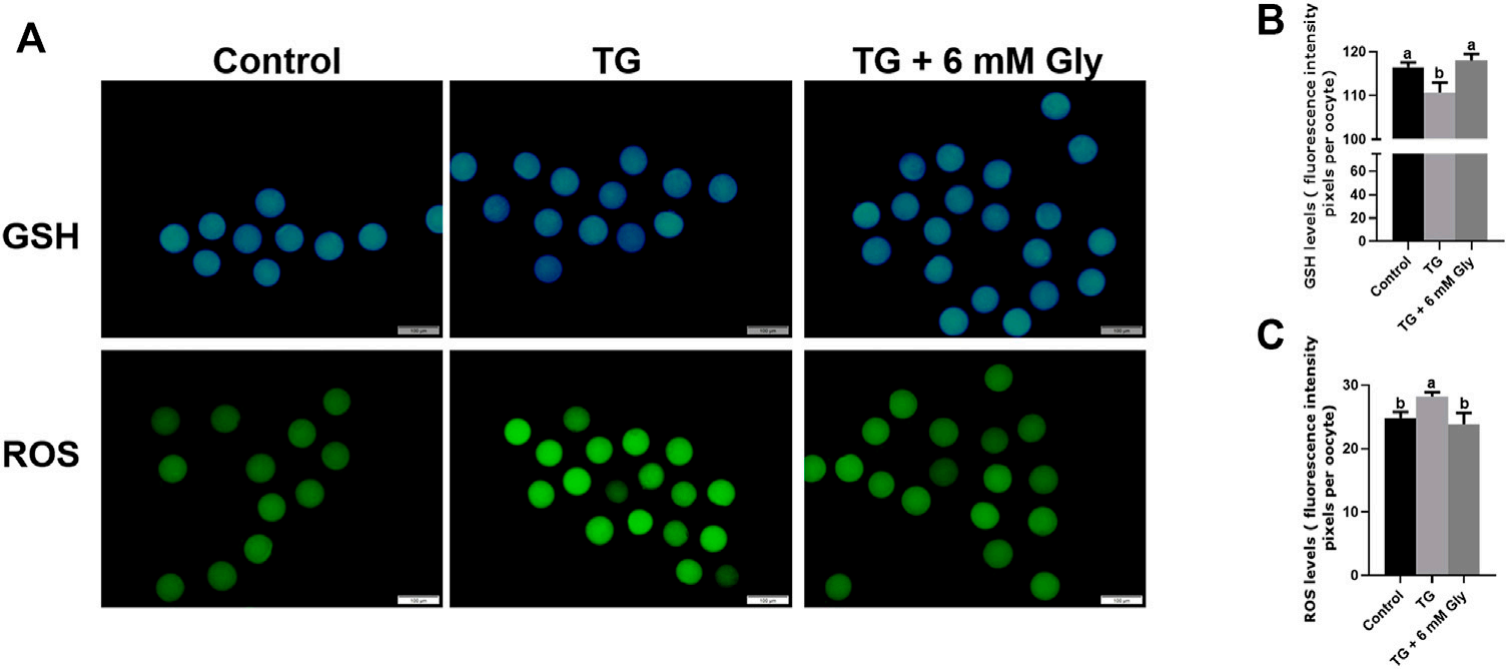
Effect of Gly on the GSH and ROS levels in porcine oocytes treated with TG. **(A)** Immunofluorescent staining of GSH (blue) and ROS (green) in MII porcine oocytes. Bar = 200 µm. **(B, C)** Fluorescence intensity analysis of the GSH or ROS signals by ImageJ software. Different letters above the bars represent significant differences (^a, b^ *p* < 0.05). Number of oocytes per group, GSH levels (Control = 45; TG = 30; TG + 6 mM Gly = 40), ROS levels (Control = 30; TG = 32; TG + 6 mM Gly = 38).

ER stress influences mitochondrial function. ROS are an index of the oxidative stress in mitochondria. To determine whether TG could induce oxidative stress in oocytes and whether Gly could alleviate this situation, we detected the GSH and ROS levels in porcine oocytes after TG treatment and Gly supplementation (Figure 2A). The GSH levels of the 50 nM TG group were significantly lower than those of the control group (*p* < 0.05). After adding Gly, the level of GSH returned to that of the control group level in porcine oocytes subjected to IVM (Figure 2B). In contrast, the ROS levels were significantly higher in the TG group. However, the addition of Gly can downregulate the level of ROS and the increase in ROS caused by ER stress induced by TG (Figure 2C) (*p* < 0.05).

### Effect of Gly on Mitochondrial Function in Porcine Oocytes Treated With TG

**FIGURE 3:**
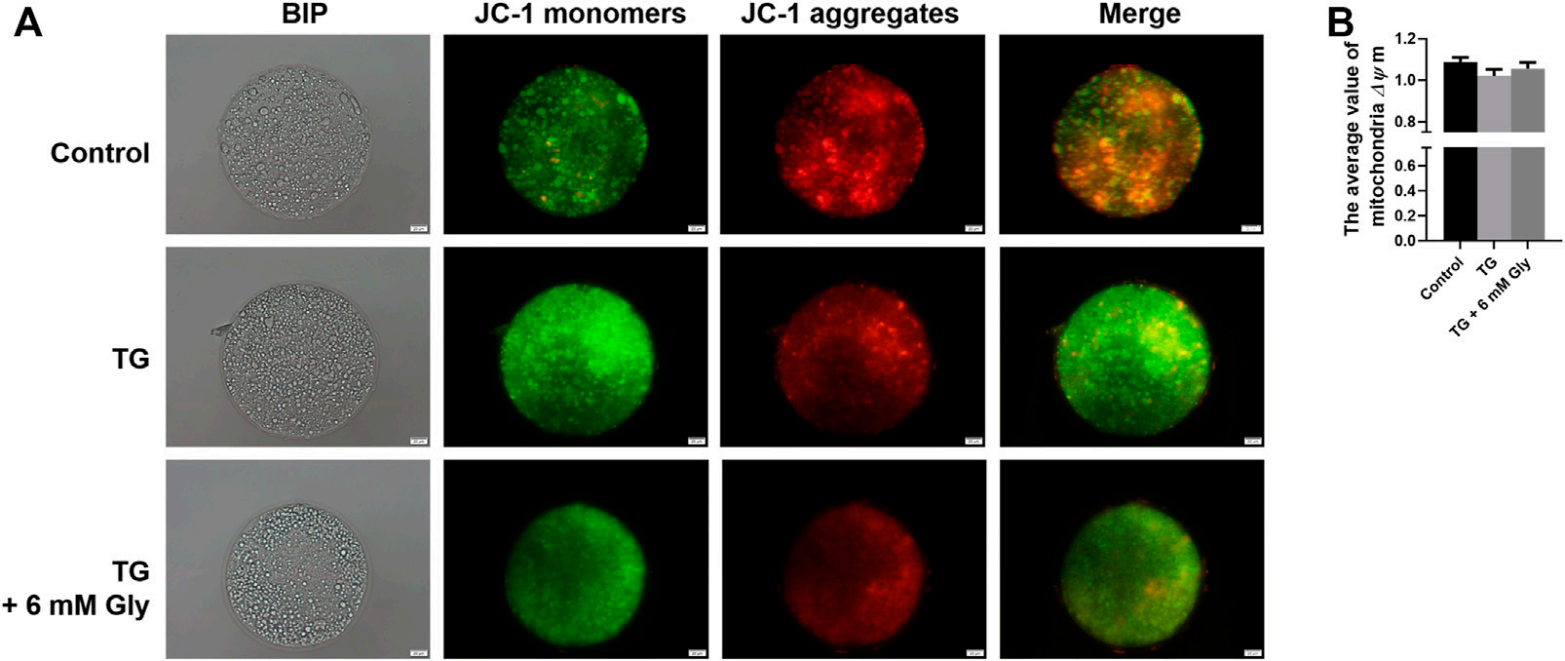
Effect of Gly on the mitochondrial membrane potential in porcine oocytes treated with TG. **(A)** In cells with a high mitochondrial ΔΨ m, J-aggregates fluoresced red, while in cells with a low mitochondrial ΔΨ m, the JC-1 monomer fluoresced green. The value of the mitochondrial ΔΨ m was expressed as the ratio of red fluorescence intensity to green fluorescence intensity. **(B)** The relative fluorescence intensity of the mitochondrial membrane potential in different TG or Gly treatment groups (^a, b^ *p* < 0.05). Number of oocytes per group (Control = 38; TG = 33; TG + 6 mM Gly = 39).

We measured the mitochondrial ΔΨ m, which is an indicator of mitochondrial function in porcine oocytes. Because ER stress may provoke mitochondrial dysfunction. Plot (Figures 3A,B) shows that ΔΨ m was significantly affected in the culture group with only TG added, and the addition of Gly rescued the significant decrease in mitochondrial ΔΨ m caused by ER stress (*p* < 0.05). These results indicated that the addition of Gly could alleviate mitochondrial dysfunction, which was triggered by TG-induced ER stress.

### Effect of Gly on ER Function in Porcine Oocytes Treated With TG

**FIGURE 4:**
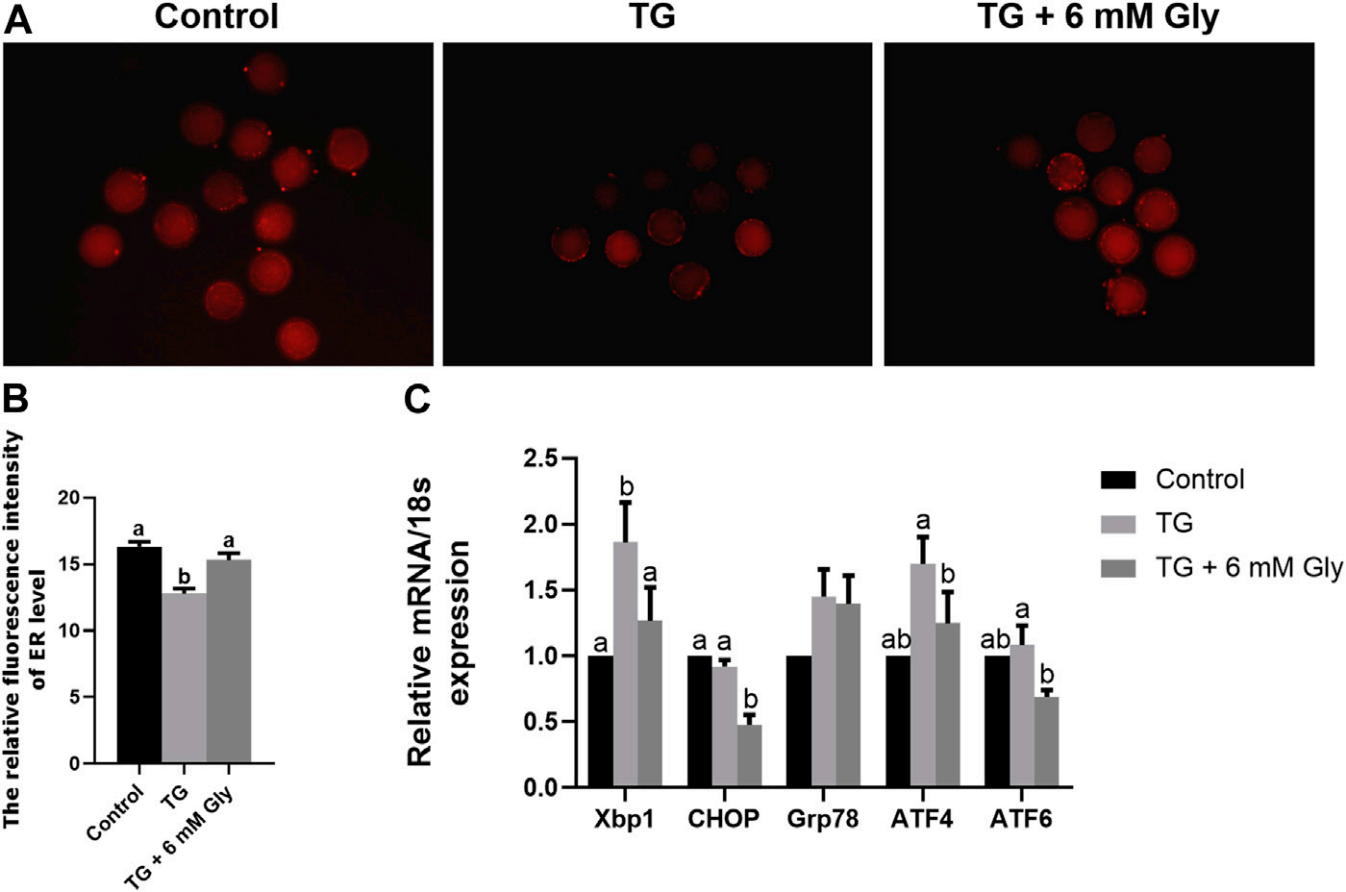
Effect of Gly on the ER levels in porcine oocytes treated with TG. **(A, B)** ER-Tracker Red (Red) signals in MII porcine oocytes. Bar = 200 μm; Number of oocytes per group (Control = 31; TG = 34; TG + 6 mM Gly = 36). **(C)** Total RNA was extracted from COCs, and the expression of the ER stress marker genes Xbp1, CHOP, Grp78, ATF4, and ATF6 was determined by real-time PCR. (^a, b^ *p* < 0.05).

In fact, the addition of Gly alone significantly increased the level of ER in oocytes compared with the blank control group. In ER stress related genes, the results showed that CHOP, Xbp1, and ATF4 were significantly decreased in mature oocytes after Gly treatment (Supplementary Figure S3). After determining the role of Gly, to detected the effects of Gly on ER function in oocytes in exposure to TG, we tested the level of ER in cells by ER-Tracker Red. Compared with the control group and Gly group, the ER levels showed an obvious downward trend in the TG group (*p* < 0.05). However, the addition of Gly successfully improved the TG-induced decrease in ER levels (Figures 4A,B). To further confirm the effect of Gly on ER function, we detected the mRNA expression of typical genes associated with ER stress by qPCR. As shown in the results, Bip/Grp78, ATF4, ATF6, Xbp1, and CHOP were significantly increased in oocytes that received TG relative to the control oocytes. We then examined those putative central genes in the Gly treatment group, and the mRNA expression levels of UPR signalling pathway genes were normal (Figure 4C) (*p* < 0.05). Real-time PCR analysis revealed the induction of ER stress by TG, which increased the expression of most UPR marker genes. We hypothesized that there was a protective role of Gly on TG-induced ER stress. Based on these results, we determined that porcine COCs at 44 h would be used for subsequent experiments.

### Effect of Gly on the Ca^2+^ Levels in Porcine Oocytes Treated With TG

**FIGURE 5:**
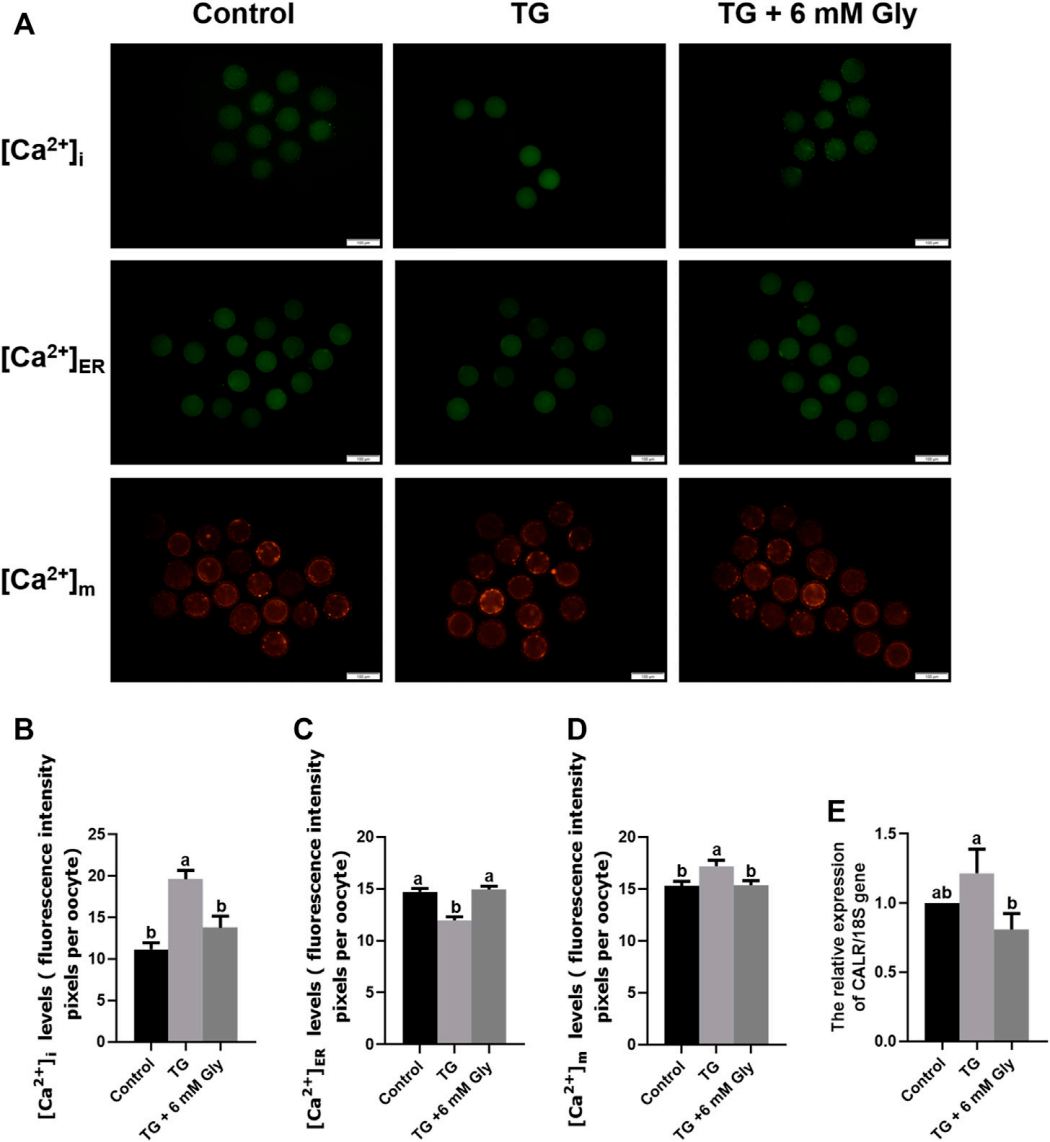
Effect of Gly on the Ca^2+^ levels in porcine oocytes treated with TG. **(A–D)** The [Ca^2+^]_i_ levels in MII porcine oocytes were detected by immunostaining with 5 μM Fluo-3/AM (green); the [Ca^2+^]_ER_ levels were detected by immunostaining with 10 μM Mag-Fluo-4 AM (green); and the [Ca^2+^]_m_ levels were detected by immunostaining with 10 μM Rhod-2 AM (orange). The levels were all measured by ImageJ software. Number of oocytes per group, [Ca^2+^]_i_ (Control = 30; TG = 34; TG + 6 mM Gly = 35), [Ca^2+^]_m_ (Control = 51; TG = 43; TG + 6 mM Gly = 37), [Ca^2+^]_ER_ (Control = 34; TG = 30; TG + 6 mM Gly = 38). **(E)** Total RNA was extracted from COCs, and the expression of the Ca^2+^-related gene CALR was determined by real-time PCR (^a, b^ *p* < 0.05).

As shown in Figure 5A, we tested the Ca^2+^ levels in porcine oocytes, including [Ca^2+^]_i_ levels, [Ca^2+^]_ER_ levels and [Ca^2+^]_m_ levels. Our Fluo-3AM (5 μM) fluorescent staining evidenced that the level of [Ca^2+^]_i_ in the TG group was visibly higher than those of other groups (*p* < 0.05), reaching almost twice as high as the control group. In the TG + 6 mM Gly group, although the level of [Ca^2+^]_i_ was still higher than that in the control group, it was not significantly different from that in the control group (Figure 5B).

Importantly, to detect the whole dynamic balance of calcium ions in cells, we also stained the calcium ions in the ER and mitochondria with specific probes. The results showed that the trend for the mitochondrial calcium changes in the three groups was the same as that of [Ca^2+^]_i_ levels, while the calcium in the ER was opposite to the first two. The [Ca^2+^]_ER_ in the TG group decreased significantly, while the calcium ions in the control group and Gly group were significantly higher than those in the TG group (Figure 5C). In addition, we found that the levels of [Ca^2+^]_i_ and [Ca^2+^]_m_ were opposite to the mitochondrial ΔΨ m levels. In the TG group, the levels of [Ca^2+^]_i_ and [Ca^2+^]_m_ were increased significantly (Figure 5D), while the mitochondrial ΔΨ m of mitochondria decreased significantly, indicating that a high level of [Ca^2+^]_i_ was not conducive to the normal function of mitochondria in oocytes. In addition, to further determine the changes in calcium ion levels, we detected calcium ion-related genes by qPCR. The mRNA expression of calreticulin (CALR) was also significantly increased in the TG group (Figure 5E). To maintain the calcium ion balance in cells, [Ca^2+^]_ER_ may flow out violently after ER stress, leading to an increase in the calcium concentration in the cytoplasm and mitochondria. The results showed that in TG-induced ER stress, the addition of Gly could effectively regulate the dynamic balance of calcium, ameliorate the excessive [Ca^2+^]_i_ and [Ca^2+^]_m_ caused by ER stress, and inhibit [Ca^2+^]_ER_ outflow. Adding Gly could improve mitochondrial dysfunction and promote oocyte maturation. Therefore, the addition of 6 mM Gly could effectively change oocyte Ca^2+^ levels, which were increased by TG.

In order to confirm the regulatory effect of Gly on calcium ion, Gly was added separately and compared with the blank control group. The results showed that added Gly could decrease the [Ca^2+^]_i_ and [Ca^2+^]_m_ but could increase the [Ca^2+^]_ER_, so Gly could affect calcium levels (Supplementary Figure S4).

### Effect of Gly on Apoptosis in Porcine Oocytes Treated With TG

**FIGURE 6:**
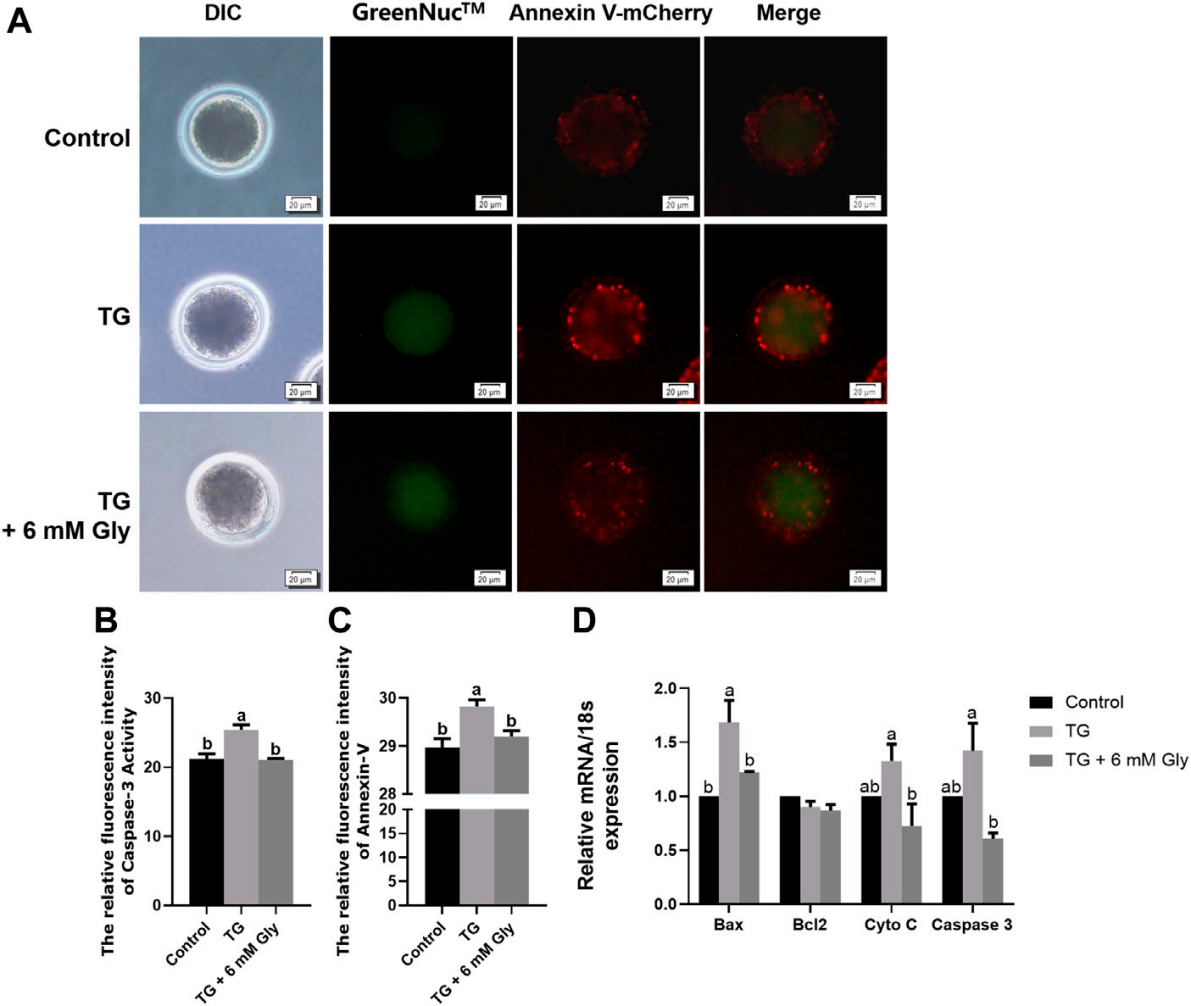
Effect of Gly on apoptosis in porcine oocytes treated with TG. **(A)** Caspase 3 activity (GreenNuc™, green) and Annexin-V signals (Annexin V-mCherry, Red) in MII porcine oocytes. Bar = 20 µm. **(B)** Fluorescence intensity analysis of Caspase 3 activity by ImageJ software. **(C)** Fluorescence intensity analysis of the Annexin-V signals by ImageJ software. Number of oocytes per group (Control = 35; TG = 30; TG + 6 mM Gly = 35). **(D)** Total RNA was extracted from COCs, and the expression of the apoptosis-related genes Bax, Bcl2, Cyto C, and Caspase 3 was determined by real-time PCR (^a, b^ *p* < 0.05).

Since apoptosis will be induced by some stress reactions, we then examined the activity of the apoptosis-associated protein Caspase 3 and Annexin-V signals to prove whether ER stress induced by TG could cause apoptosis in oocytes *in vitro*. As shown in the results, the almost invisible Caspase 3 expression and Annexin-V signals were very weak in the control group. TG treatment greatly increased Caspase 3 expression and Annexin-V signals in comparison with controls levels of fluorescence intensity (Figure 6A). TG-treated oocytes showed clear positive Caspase 3 and Annexin-V signals, indicating the occurrence of apoptosis. Remarkably, we found that adding Gly altered this phenomenon, and the Caspase 3 activity and Annexin-V signals were normalized in oocytes from the TG + 6 mM Gly group (Figures 6B,C).

On the other hand, to improve the accuracy of apoptosis detection caused by ER stress induced by TG, we evaluated the mRNA expression of genes related to apoptosis in mature oocytes. As shown in the results, Bax, Cyto C, and Caspase 3 were significantly upregulated, but the Bcl2 gene was downregulated in the TG group compared to the control group (*p* < 0.05). Conversely, the expression of proapoptotic genes was obviously decreased after Gly treatment (Figure 6D). These data indicate the protective role of Gly against TG-induced apoptosis.

### Effect of Gly on the IP_3_R1 and VDAC1 Cellular Distribution in Porcine Oocytes Treated With TG

**FIGURE 7:**
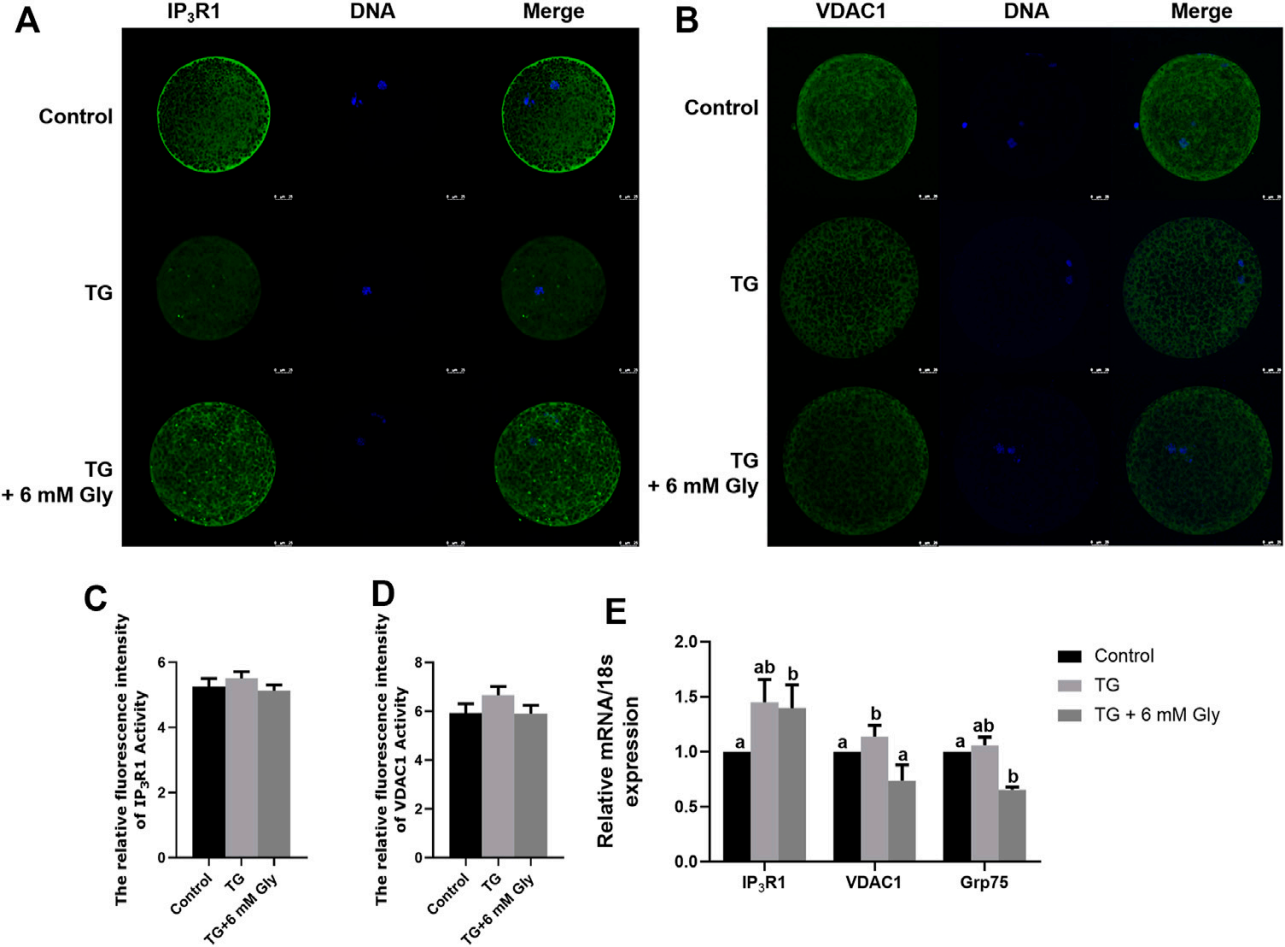
Effect of Gly on the IP_3_R1 and VDAC1 cellular distributions in porcine oocytes treated with TG. **(A, C)** IP_3_R1 **c**ellular distribution pattern after TG treatment or Gly supplementation. IP_3_R1 (green), DNA (blue), and merged images in MII oocytes. **(B, D)** VDAC1 cellular distribution pattern after TG treatment or Gly supplementation. VDAC1 (green), DNA (blue), and merged images in MII oocytes. Number of oocytes per group, IP_3_R1 (Control = 42; TG = 43; TG + 6 mM Gly = 49), VDAC1 (Control = 49; TG = 46; TG + 6 mM Gly = 40). **(E)** Total RNA was extracted from COCs, and the expression of the IP_3_R1, VDAC1 and Grp75 was determined by real-time PCR (^a, b^ *p* < 0.05).

In maintaining intracellular calcium homeostasis, the IP_3_R1 and VDAC1 proteins are important proteins. Our results showed that Gly inhibited the expression of IP_3_R protein and the mRNA levels of IP_3_R1 (Supplementary Figure S5) and promoted the aggregation of cells in the cortex. In order to deepen the study of the effects of Gly on calcium signalling pathway in oocytes in exposure to TG, we tested the localization of IP_3_R1 and VDAC1. Normally, IP_3_R1 and VDAC1 were distributed in the cortical portion in the control group. Nevertheless, the regular distribution of IP_3_R1 and VDAC1 was disturbed, and they were observed in the cytoplasm in oocytes in the TG group. Here, we showed that TG-induced ER stress led to the cortical redistribution of IP_3_R1 and VDAC1, as oocytes that matured in the presence of TG lacked IP_3_R1 and VDAC1 cortical clusters. These proteins were evenly distributed in the cytoplasm rather than accumulated in the cortex. Furthermore, the distribution patterns of IP_3_R1 and VDAC1 in the Gly group redistributed into the cortical portion, similar to those of the controls (Figures 7A–D). The qPCR analysis further verified that the mRNA expression of IP_3_R1, VDAC1 and Grp75 was significantly increased in the TG group (Figure 7E) (*p* < 0.05). Their change trends showed consistent results.

### Effects of TG and Gly on Embryonic Development After PA

**FIGURE 8:**
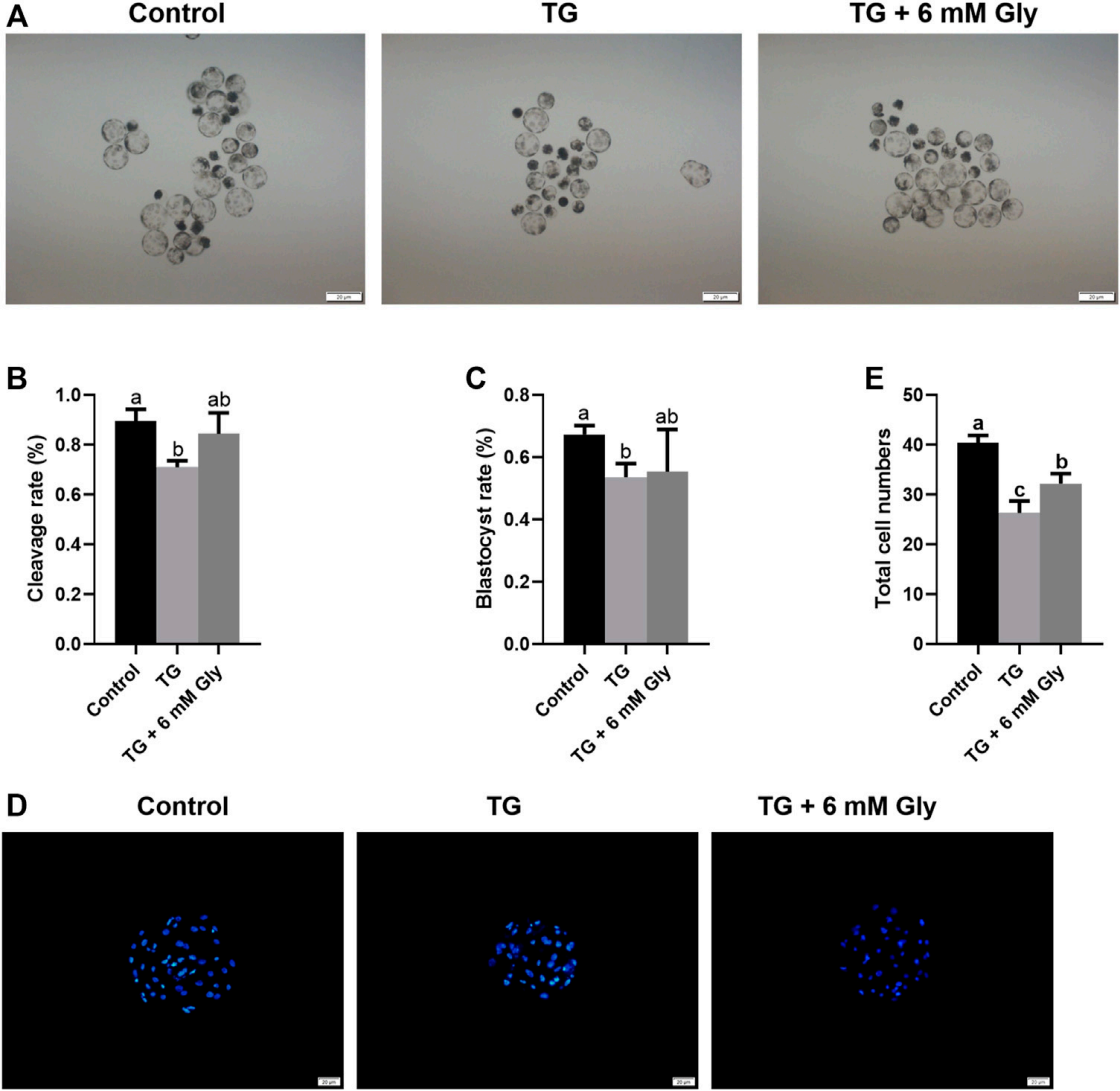
Effects of TG and Gly on embryonic development after PA. Significant differences are represented with different letters (^a, b, c^ *p* < 0.05). **(A)** Porcine PA blastocysts induced from different treatment groups (100× magnification). **(B)** Cleavage rate of embryos in different treatment groups. **(C)** Blastocyst rate of embryos in different treatment groups. **(D)** Hoechst 33,342 staining (blue) of blastocysts on day 7 (200× magnification) in different treatment groups. **(E)** Total cell numbers in blastocysts on day 7 in the different treatment groups. Number of oocytes per group, cleavage rate (Control = 112; TG = 97; TG + 6 mM Gly = 94), blastocyst rate (Control = 73; TG = 63; TG + 6 mM Gly = 70).

To detect the early embryonic development of pigs treated with TG or Gly, MII oocytes were parthenogenetically activated. From the development morphology of blastocysts, the blastocysts in the TG group were significantly smaller than those in the other two groups (Figure 8A). The statistical results showed that there was an apparent decline in the cleavage rate in the TG group, but Gly reduced this damage, and the cleavage rate even reached the level of the control group (89.61 vs. 71.03% and 84.43%, *p* < 0.05) (Figure 8B). The blastocyst rate also showed the same result in which adding 6 mM Gly increased the rate to a level that was higher than that in the TG group but not the control group (67.27 vs. 53.59% and 55.4%, *p* < 0.05) (Figure 8C). And the total cell numbers shown the results consistent with the above two indicators (Figures 8D,E). Based on these results, we hypothesized that adding 6 mM Gly could ameliorate ER stress and was beneficial to oocyte IVM and the subsequent development of PA embryos.

## Discussion

In this study, we aimed to investigate the regulatory effect of Gly on ER stress induced by calcium homeostasis imbalance in porcine oocytes during IVM. The regulation and effect of Gly on ER stress during IVM of porcine oocytes were first explored by detecting key factors of the UPR signalling pathway; ER and mitochondrial function; the [Ca^2+^]_i_, [Ca^2+^]_m_ and [Ca^2+^]_ER_ levels; the transport channels IP_3_R1 and VDAC1; and apoptosis. Gly reduced the TG-induced defects in ER stress.

According to a previous study, adding antioxidants and Gly could improve the developmental competence of vitrified-warmed ovine immature oocytes, especially when added during IVM (Ahmadi et al., 2019). Similar to our results, adding Gly improved the porcine oocyte maturation rate and blastocyst rate.

TG is a drug that is known to induce ER stress and to increase the expression of UPR marker genes after ER stress inducer treatment during IVM (Gu et al., 2016). ATF4, ATF6, Xbp1, and Grp78 are ER stress-related genes. Variation in Xbp1 splicing is extensively used to monitor ER stress both *in vivo* and *in vitro* in mouse oocytes (Iwawaki et al., 2004). One study reported that ER stress was induced by the ER Ca^2+^ channel blocker TG, which increased the expression of ATF4, ATF6, Xbp1, and Grp78 in mouse COCs during IVM (Wu et al., 2012). Our results also showed that the mRNA expression of Xbp1, CHOP, ATF4 and ATF6 was significantly increased and that ER function was significantly decreased after TG treatment. Gly treatment greatly decreased the expression of Xbp1, CHOP, ATF4, and ATF6 in porcine oocytes. Therefore, as the results showed that Gly could regulate ER function and modulate ER stress.

The ER is the main storage organelle of Ca^2+^, and mitochondria are the main effectors that regulate Ca^2+^ uptake and intrinsic apoptosis in cells. The dynamic balance of Ca^2+^ plays an important role in maintaining organelle function and homeostasis. ER stress could lead to an increase in the [Ca^2+^]_i_ induced by Ca^2+^ transfer from the ER cavity to the cytoplasm. The increasing [Ca^2+^]_i_ is an important reason for mitochondrial dysfunction. In fact, our results illustrated that [Ca^2+^]_i_ levels were significantly elevated in porcine oocytes after TG treatment. At the same time, when the [Ca^2+^]_i_ level increased, the mitochondrial ΔΨ m began to decrease the [Ca^2+^]_i_ (Bustos et al., 2017; Missiroli et al., 2017; Prudent and McBride, 2017; Marchi et al., 2018). The level of calcium ions in the ER decreased significantly in the TG group. Based on the principle of intracellular calcium ion dynamic balance, we think that after ER stress, calcium ions in the ER continue to be released outward, resulting in a surge of calcium ions in the cytoplasm. Further evidence at the gene level also confirms this result. The mRNA expression of CALR, the gene of a key ER calcium binding protein, was significantly increased. In addition, we found that when the intracellular calcium ion levels increased, the calcium ion levels in mitochondria increased significantly, but the mitochondrial ΔΨ m decreased significantly in the TG group. This result indicates that the high concentration of calcium in mitochondria will lead to damage to mitochondrial function. However, after the addition of Gly, the [Ca^2+^]_i_, [Ca^2+^]_m_ and [Ca^2+^]_ER_ levels were rescued, and the mitochondrial ΔΨ m was also improved, which is consistent with the previous conclusion that reducing mitochondrial Ca^2+^ levels can improve mitochondrial function (Zhang et al., 2020).

Several studies have shown that the protective effect of Gly is achieved by reducing intracellular Ca^2+^ overload (Qu et al., 2002). These results support the hypothesis that Gly reverses the ER stress induced by TG and improves the developmental competence of oocytes by regulating intracellular Ca^2+^ levels.

Previous studies have demonstrated that excessive ROS are produced when mitochondrial dysfunction leads to oxidative stress and cell damage (Bhatti et al., 2017). However, ROS and Ca^2+^ signals regulate each other. Ca^2+^ overload of the mitochondrial matrix can lead to an increase in ROS, trigger permeability transition pores, release Cyto C, and lead to apoptosis (Brookes et al., 2004). Therefore, we measured ROS levels, and our results showed that ROS levels were significantly increased in the TG group. In addition, the dynamic changes between ROS and GSH are related to oocyte competence (Barros et al., 2019). We also measured GSH levels in all groups, and the results showed that the GSH levels were significantly decreased in the TG group. Gly is the main component of GSH. Our previous study also verified that Gly treatment had a beneficial effect on *in vitro* oocyte maturation and blastocyst development by decreasing ROS levels to reduce apoptosis (Qu et al., 2002; Yu et al., 2021). In this study, Gly treatment reversed the decrease in GSH levels and the increase in ROS levels induced by TG. It has been reported that the mitochondrial respiratory chain in oocytes caused by high levels of ROS is mediated by mitochondrial Ca^2+^ levels; this is consistent with our results (Zhang et al., 2020).

ER stress, mitochondrial dysfunction, and an increase in [Ca^2+^]_i_ or ROS levels can cause apoptosis. ER stress can increase the expression of CHOP in the UPR. CHOP can trigger the intrinsic apoptotic pathway through the inhibition of BCL2, BCL-XL, and MCL-1 and the upregulation of BIM, which regulates BAX-BAK-mediated mitochondrial outer membrane permeabilization, leading to Cyto C release and the Caspase cascade (Hu et al., 2018; Kim and Kim, 2018). Previous studies have identified that the expression of key regulators of oocyte apoptosis (Bax and Caspase 3) is increased in mouse oocyte apoptosis (Escobar et al., 2019; Wang F. et al., 2020; Wang L. et al., 2020). Overexpression of Bax modulates the mitochondrial ΔΨ m and triggers Cyto C release and induces the activation of Caspase 3, leading to DNA fragmentation and thereby oocyte apoptosis (Chaube et al., 2014). Furthermore, Gly addition during both vitrification/thawing and maturation enhanced the oocyte quality, as demonstrated by the anti-apoptosis effects in mouse oocytes (Anchordoquy et al., 2019). Therefore, we examined Caspase 3 activity, Annexin-V signals and the expression of apoptosis-related genes, such as Bax, Bcl2, Caspase 3, and Cyto C. The results indicated that Caspase 3 activity and Annexin-V signals were significantly increased in the TG group, and the mRNA expression of Bax, Caspase 3 and Cyto C was significantly increased after TG treatment. Thus, Gly ameliorates the overexpression of apoptotic proteins and genes.

IP_3_R and VDAC1 are the main channels involved in MAM Ca^2+^ transport. IP_3_R1 is located in the ER membrane, VDAC1 is located in the mitochondrial membrane, and IP_3_R connects with VDAC1 to control Ca^2+^ release from the ER to mitochondria and the cytoplasm (Moltedo et al., 2019). The Ca^2+^ stored in the ER is released through IP_3_R, diffuses through the MAMs, is absorbed by VDAC1, and is transported to the mitochondrial matrix by MCU. Some studies have shown that increasing the [Ca^2+^]_i_ through IP_3_R induces ER stress and inhibits the UPR signalling pathway. Excessive Ca^2+^ influx into mitochondria causes the opening of mitochondrial permeability transition pores, resulting in the production of ROS, and leads to the release of Cyto C, which can not only affect the developmental potential of oocytes but can also induce cell apoptosis. Knockdown of IP_3_R1 expression in mice could improve mitochondrial function and alleviate oocyte damage caused by obesity (L. Zhao et al., 2017). At present, two main pathways by which Gly prevents calcium overload have been reported: the binding of Gly with its receptor GlyR activates PKC and increases IP_3_, which activates the IP_3_R to increase intracellular Ca^2+^ release. Additionally, the binding of Gly with GlyR leads to membrane hyperpolarization and decreases the opening of voltage-dependent Ca^2+^ channels (such as VDAC). Gly could reduce the extracellular Ca^2+^ influx and alleviate Ca^2+^ overload, which ameliorates mitochondrial dysfunction and reduces apoptosis (Qu et al., 2002; Van den Eynden et al., 2009; M.; Wheeler et al., 2000; M. D.; Wheeler et al., 1999; Yamashina et al., 2001). Therefore, we examined the cellular distribution and localization pattern of IP_3_R1 and VDAC1 during porcine oocyte maturation. IP3R1 and VDAC1 have been reported to be distributed in the cortex of oocytes and to form cortical clusters. Our study produced similar results, and we observed that IP_3_R1 and VDAC1 in porcine oocytes were distributed in the cytoplasm and formed cortical clusters in the control group. However, the distribution and mRNA expression of IP_3_R1 or VDAC1 were changed after TG treatment. Both IP_3_R1 and VDAC1 were transferred to and distributed in the cytoplasm when porcine oocytes were exposed to TG. The mRNA expression of IP_3_R1 and VDAC1 was significantly increased in the TG group. Fortunately, the distribution and mRNA expression of IP_3_R1 and VDAC1 were recovered to the control level after Gly treatment. Our results demonstrate that VDAC1 selectively interacts with IP_3_Rs and plays a key role in the processes by which the ER transmits Ca^2+^ to mitochondria, affecting the function of the ER and mitochondria. It has been reported that there is a connexin Grp75 between IP_3_R and the VDAC1 pathway (Mendes et al., 2005; Szabadkai et al., 2006), and researchers have found that calcium transport in cells can be regulated through the IP_3_R-Grp75-VDAC1 pathway. IP_3_R1 is the most widely expressed subtype in mammals. Therefore, in this experiment, we detected the IP_3_R-Grp75-VDAC1 pathway. The results show that calcium ions are likely to be regulated by this pathway during porcine oocyte IVM, which is similar to previous results.

Finally, we tested whether early embryo development was affected by TG or Gly treatment. Compared with those of the control group, the TG group had a low cleavage rate, blastocyst rate, and cell number. However, treatment with Gly altered the effects of TG. An increasing number of studies have indicated that mammalian oocyte maturation and early embryo development processes are Ca^2+^-dependent. There are specific distribution patterns and dynamic changes in Ca^2+^ that take place during the processes of bovine oocyte maturation and PA embryo development *in vitro* (Liang et al., 2011). Therefore, the addition of Gly at the IVM stage is beneficial to the development of an early embryo after PA.

## Summary

**FIGURE 9:**
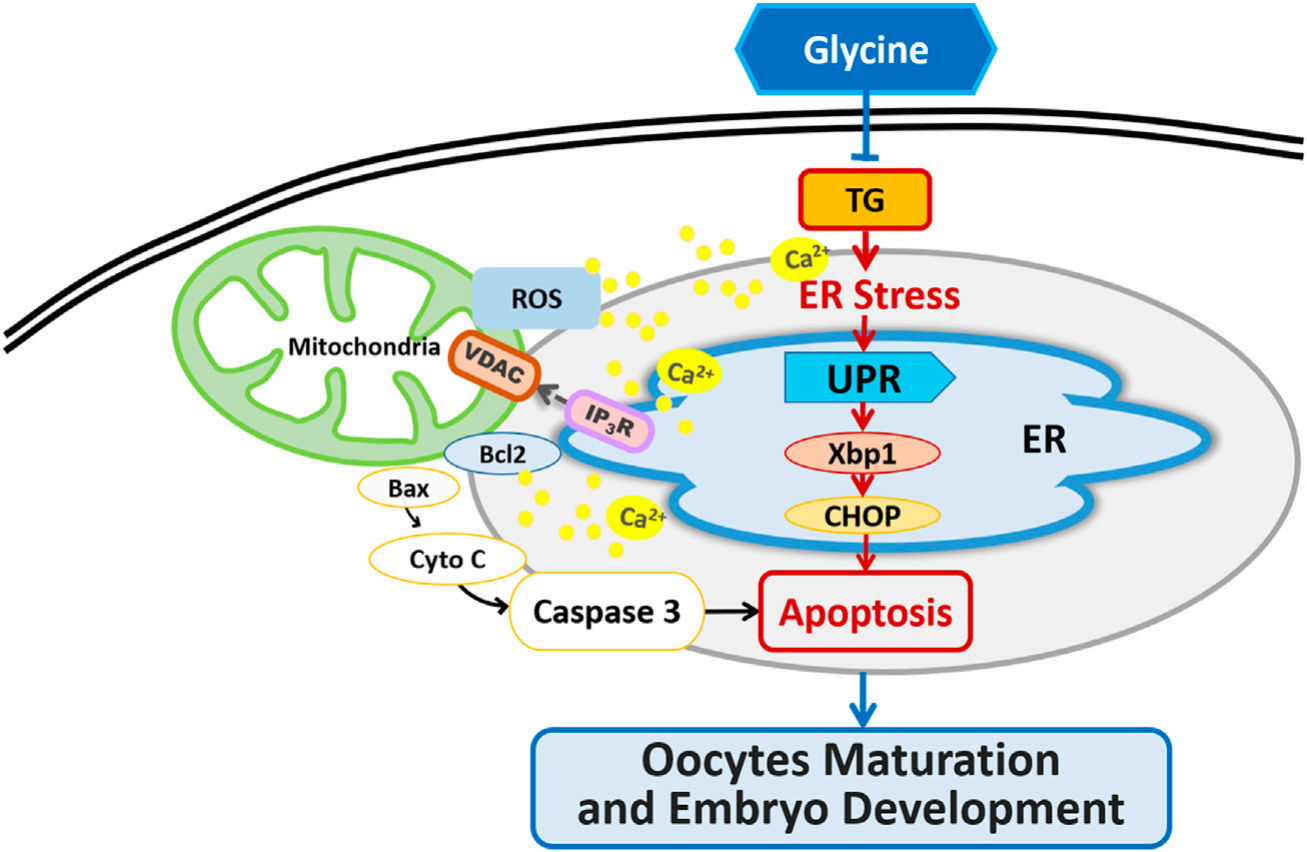
Graphical abstract of how Gly protects porcine oocyte maturation against the effects of TG treatment.

We concluded that Gly could ameliorate ER stress and apoptosis in TG-exposed porcine oocytes by decreasing UPR and apoptosis-related gene expression, regulating [Ca^2+^]_i_ levels, restoring the distribution and mRNA expression of IP_3_R1 and VDAC1 and further enhancing the developmental potential of porcine oocytes *in vitro* (Figure 9)*.*

## Data Availability

The original contributions presented in the study are included in the article/Supplementary Material, further inquiries can be directed to the corresponding authors.

## References

[B1] Ahmadi E. Shirazi A. Shams‐Esfandabadi N. Nazari H. (2019). Antioxidants and glycine Can Improve the Developmental Competence of Vitrified/warmed Ovine Immature Oocytes. Reprod. Dom Anim. 54 (3), 595–603. 10.1111/rda.13402 30637807

[B2] Anchordoquy J. P. Lizarraga R. M. Anchordoquy J. M. Nikoloff N. Rosa D. E. Fabra M. C. (2019). Effect of Cysteine, Glutamate and glycine Supplementation to *In Vitro* Fertilization Medium during Bovine Early Embryo Development. Reprod. Biol. 19 (4), 349–355. 10.1016/j.repbio.2019.10.002 31722857

[B3] Ando H. Kawaai K. Bonneau B. Mikoshiba K. (2018). Remodeling of Ca 2+ Signaling in Cancer: Regulation of Inositol 1,4,5-trisphosphate Receptors through Oncogenes and Tumor Suppressors. Adv. Biol. Regul. 68, 64–76. 10.1016/j.jbior.2017.12.001 29287955

[B4] Barros F. D. d. A. Adona P. R. Guemra S. Damião B. C. M. (2019). Oxidative Homeostasis in Oocyte Competence for *In Vitro* Embryo Development. Anim. Sci. J. 90 (10), 1343–1349. 10.1111/asj.13256 31469477

[B5] Bhatti J. S. Bhatti G. K. Reddy P. H. (2017). Mitochondrial Dysfunction and Oxidative Stress in Metabolic Disorders - A Step towards Mitochondria Based Therapeutic Strategies. Biochim. Biophys. Acta (Bba) - Mol. Basis Dis. 1863 (5), 1066–1077. 10.1016/j.bbadis.2016.11.010 PMC5423868 27836629

[B6] Bootman M. D. Collins T. J. Peppiatt C. M. Prothero L. S. MacKenzie L. De Smet P. (2001). Calcium Signalling-An Overview. Semin. Cel Develop. Biol. 12 (1), 3–10. 10.1006/scdb.2000.0211 11162741

[B7] Brookes P. S. Yoon Y. Robotham J. L. Anders M. W. Sheu S.-S. (2004). Calcium, ATP, and ROS: a Mitochondrial Love-Hate triangle. Am. J. Physiology-Cell Physiol. 287 (4), C817–C833. 10.1152/ajpcell.00139.2004 15355853

[B8] Bustos G. Cruz P. Lovy A. Cárdenas C. (2017). Endoplasmic Reticulum-Mitochondria Calcium Communication and the Regulation of Mitochondrial Metabolism in Cancer: A Novel Potential Target. Front. Oncol. 7, 199. 10.3389/fonc.2017.00199 28944215 PMC5596064

[B9] Chaube S. K. Shrivastav T. G. Tiwari M. Prasad S. Tripathi A. Pandey A. K. (2014). Neem (Azadirachta indica L.) Leaf Extract Deteriorates Oocyte Quality by Inducing ROS-Mediated Apoptosis in Mammals. Springerplus 3, 464. 10.1186/2193-1801-3-464 25197620 PMC4155053

[B10] Chevet E. Cameron P. H. Pelletier M. F. Thomas D. Y. Bergeron J. J. M. (2001). The Endoplasmic Reticulum: Integration of Protein Folding, Quality Control, Signaling and Degradation. Curr. Opin. Struct. Biol. 11 (1), 120–124. 10.1016/s0959-440x(00)00168-8 11179901

[B11] Escobar M. L. Echeverria O. M. Palacios-Martínez S. Juárez-Chavero S. Sánchez-Sánchez L. Vázquez-Nin G. H. (2019). Beclin 1 Interacts with Active Caspase-3 and Bax in Oocytes from Atretic Follicles in the Rat Ovary. J. Histochem. Cytochem. 67 (12), 873–889. 10.1369/0022155419881127 31583941 PMC6882064

[B12] Fung T. S. Liu D. X. (2014). Coronavirus Infection, ER Stress, Apoptosis and Innate Immunity. Front. Microbiol. 5, 296. 10.3389/fmicb.2014.00296 24987391 PMC4060729

[B13] Groenendyk J. Michalak M. (2005). Endoplasmic Reticulum Quality Control and Apoptosis. Acta Biochim. Pol. 52 (2), 381–395. 10.18388/abp.2005_3451 15933766

[B14] Gu X.-W. Yan J.-Q. Dou H.-T. Liu J. Liu L. Zhao M.-L. (2016). Endoplasmic Reticulum Stress in Mouse Decidua during Early Pregnancy. Mol. Cell Endocrinol. 434, 48–56. 10.1016/j.mce.2016.06.012 27283502

[B15] Hitomi J. Katayama T. Eguchi Y. Kudo T. Taniguchi M. Koyama Y. (2004). Involvement of Caspase-4 in Endoplasmic Reticulum Stress-Induced Apoptosis and Aβ-Induced Cell Death. J. Cel Biol 165 (3), 347–356. 10.1083/jcb.200310015 PMC2172196 15123740

[B16] Hu H. Tian M. Ding C. Yu S. (2018). The C/EBP Homologous Protein (CHOP) Transcription Factor Functions in Endoplasmic Reticulum Stress-Induced Apoptosis and Microbial Infection. Front. Immunol. 9, 3083. 10.3389/fimmu.2018.03083 30662442 PMC6328441

[B17] Iwawaki T. Akai R. Kohno K. Miura M. (2004). A Transgenic Mouse Model for Monitoring Endoplasmic Reticulum Stress. Nat. Med. 10 (1), 98–102. 10.1038/nm970 14702639

[B18] Jheng J.-R. Ho J.-Y. Horng J.-T. (2014). ER Stress, Autophagy, and RNA Viruses. Front. Microbiol. 5, 388. 10.3389/fmicb.2014.00388 25140166 PMC4122171

[B19] Khatun H. Wada Y. Konno T. Tatemoto H. Yamanaka K.-i. (2020). Endoplasmic Reticulum Stress Attenuation Promotes Bovine Oocyte Maturation *In Vitro* . Reproduction 159 (4), 361–370. 10.1530/rep-19-0492 31990669

[B20] Kim C. Kim B. (2018). Anti-Cancer Natural Products and Their Bioactive Compounds Inducing ER Stress-Mediated Apoptosis: A Review. Nutrients 10 (8), 1021. 10.3390/nu10081021 PMC6115829 30081573

[B21] Larner S. F. Hayes R. L. Wang K. K. W. (2006). Unfolded Protein Response after Neurotrauma. J. Neurotrauma 23 (6), 807–829. 10.1089/neu.2006.23.807 16774469

[B22] Lee K. Tirasophon W. Shen X. Michalak M. Prywes R. Okada T. (2002). IRE1-mediated Unconventional mRNA Splicing and S2P-Mediated ATF6 Cleavage Merge to Regulate XBP1 in Signaling the Unfolded Protein Response. Genes Dev. 16 (4), 452–466. 10.1101/gad.964702 11850408 PMC155339

[B23] Li S. Guo Q. Wang Y.-M. Li Z.-Y. Kang J.-D. Yin X.-J. (2018). Glycine Treatment Enhances Developmental Potential of Porcine Oocytes and Early Embryos by Inhibiting Apoptosis1. J. Anim. Sci. 96 (6), 2427–2437. 10.1093/jas/sky154 29762687 PMC6095358

[B24] Liang S. L. Zhao Q. J. Li X. C. Jin Y. P. Wang Y. P. Su X. H. (2011). Dynamic Analysis of Ca2+level during Bovine Oocytes Maturation and Early Embryonic Development. J. Vet. Sci. 12 (2), 133–142. 10.4142/jvs.2011.12.2.133 21586872 PMC3104167

[B25] Lin T. Lee J. E. Oqani R. K. Kim S. Y. Cho E. S. Jeong Y. D. (2016). Tauroursodeoxycholic Acid Improves Pre-implantation Development of Porcine SCNT Embryo by Endoplasmic Reticulum Stress Inhibition. Reprod. Biol. 16 (4), 269–278. 10.1016/j.repbio.2016.10.003 27765486

[B26] Marchi S. Patergnani S. Missiroli S. Morciano G. Rimessi A. Wieckowski M. R. (2018). Mitochondrial and Endoplasmic Reticulum Calcium Homeostasis and Cell Death. Cell Calcium 69, 62–72. 10.1016/j.ceca.2017.05.003 28515000

[B27] Mei Y. Thompson M. D. Cohen R. A. Tong X. (2013). Endoplasmic Reticulum Stress and Related Pathological Processes. J. Pharmacol. Biomed. Anal. 1 (2), 1000107. 10.4172/2327-4638.1000105 24611136 PMC3942890

[B28] Mendes C. C. P. Gomes D. A. Thompson M. Souto N. C. Goes T. S. Goes A. M. (2005). The Type III Inositol 1,4,5-Trisphosphate Receptor Preferentially Transmits Apoptotic Ca2+ Signals into Mitochondria. J. Biol. Chem. 280, 40892–40900. 10.1074/jbc.M506623200 16192275

[B29] Missiroli S. Danese A. Iannitti T. Patergnani S. Perrone M. Previati M. (2017). Endoplasmic Reticulum-Mitochondria Ca 2+ Crosstalk in the Control of the Tumor Cell Fate. Biochim. Biophys. Acta (Bba) - Mol. Cel Res. 1864 (6), 858–864. 10.1016/j.bbamcr.2016.12.024 28064002

[B30] Moltedo O. Remondelli P. Amodio G. (2019). The Mitochondria-Endoplasmic Reticulum Contacts and Their Critical Role in Aging and Age-Associated Diseases. Front. Cel Dev. Biol. 7, 172. 10.3389/fcell.2019.00172 PMC6712070 31497601

[B31] Prudent J. McBride H. M. (2017). The Mitochondria-Endoplasmic Reticulum Contact Sites: a Signalling Platform for Cell Death. Curr. Opin. Cel Biol. 47, 52–63. 10.1016/j.ceb.2017.03.007 28391089

[B32] Qu W. Ikejima K. Zhong Z. Waalkes M. P. Thurman R. G. (2002). Glycine Blocks the Increase in Intracellular Free Ca2+ Due to Vasoactive Mediators in Hepatic Parenchymal Cells. Am. J. Physiology-Gastrointestinal Liver Physiol. 283 (6), G1249–G1256. 10.1152/ajpgi.00197.2002 12388211

[B33] Ridlo M. R. Kim G. A. Taweechaipaisankul A. Kim E. H. Lee B. C. (2021). Zinc Supplementation Alleviates Endoplasmic Reticulum Stress during Porcine Oocyte *In Vitro* Maturation by Upregulating Zinc Transporters. J. Cel Physiol 236 (4), 2869–2880. 10.1002/jcp.30052 32944961

[B34] Santulli G. Nakashima R. Yuan Q. Marks A. R. (2017). Intracellular Calcium Release Channels: an Update. J. Physiol. 595 (10), 3041–3051. 10.1113/jp272781 28303572 PMC5430224

[B35] Siman R. Flood D. G. Thinakaran G. Neumar R. W. (2001). Endoplasmic Reticulum Stress-Induced Cysteine Protease Activation in Cortical Neurons. J. Biol. Chem. 276 (48), 44736–44743. 10.1074/jbc.M104092200 11574534

[B36] Sutton-McDowall M. L. Wu L. L. Y. Purdey M. Abell A. D. Goldys E. M. MacMillan K. L. (2016). Nonesterified Fatty Acid-Induced Endoplasmic Reticulum Stress in Cattle Cumulus Oocyte Complexes Alters Cell Metabolism and Developmental Competence1. Biol. Reprod. 94 (1), 23. 10.1095/biolreprod.115.131862 26658709

[B53] Szabadkai G. Bianchi K. Várnai P. De Stefani D. Wieckowski M. R. Cavagna D. (2006). Chaperone-Mediated Coupling of Endoplasmic Reticulum and Mitochondrial Ca^2+^ Channels. J. Cell Biol. 175 (6), 901–911. 10.1083/jcb.200608073 17178908 PMC2064700

[B37] Takehara I. Igarashi H. Kawagoe J. Matsuo K. Takahashi K. Nishi M. (2020). Impact of Endoplasmic Reticulum Stress on Oocyte Aging Mechanisms. Mol. Hum. Reprod. 26 (8), 567–575. 10.1093/molehr/gaaa040 32514562

[B38] Van den Eynden J. Ali S. S. Horwood N. Carmans S. Brône B. Hellings N. (2009). Glycine and glycine Receptor Signalling in Non-neuronal Cells. Front. Mol. Neurosci. 2, 9. 10.3389/neuro.02.009.2009 19738917 PMC2737430

[B39] Wang F. Meng T.-G. Li J. Hou Y. Luo S.-M. Schatten H. (2020a). Mitochondrial Ca2 + Is Related to Mitochondrial Activity and Dynamic Events in Mouse Oocytes. Front. Cel Dev. Biol. 8, 585932. 10.3389/fcell.2020.585932 PMC7652752 33195238

[B40] Wang L. Zhang J. Zhao C. Jia Z. Feng X. (2020b). Melatonin Reverses 10-Hydroxycamptothecin-Induced Apoptosis and Autophagy in Mouse Oocyte. Reprod. Sci. 28, 1839–1849. 10.1007/s43032-020-00359-4 33104985

[B41] Wang Y. Qi J. Yu S. Zeng X. Li Z. Liang S. (2020c). Effect of Glycine on *In Vitro* Maturation Quality of Pig Oocytes and Cumulus Cell. Chin. J. Anim. Sci. 056 (002), 88–92. 96. 10.19556/j.0258-7033.20190329-08

[B42] Wheeler M. D. Ikejema K. Enomoto N. Stacklewitz R. F. Seabra V. Zhong Z. (1999). Glycine: a New Anti-inflammatory Immunonutrient. Cell Mol. Life Sci. (Cmls) 56 (9-10), 843–856. 10.1007/s000180050030 11212343 PMC11147092

[B43] Wheeler M. Stachlewitz R. F. Yamashina S. Ikejima K. Morrow A. L. Thurman R. G. (2000). Glycine‐gated Chloride Channels in Neutrophils Attenuate Calcium Influx and Superoxide Production. FASEB j. 14 (3), 476–484. 10.1096/fasebj.14.3.476 10698962

[B44] Wu L. L. Russell D. L. Norman R. J. Robker R. L. (2012). Endoplasmic Reticulum (ER) Stress in Cumulus-Oocyte Complexes Impairs Pentraxin-3 Secretion, Mitochondrial Membrane Potential (ΔΨm), and Embryo Development. Mol. Endocrinol. 26 (4), 562–573. 10.1210/me.2011-1362 22383462 PMC5417137

[B45] Yamashina S. Konno A. Wheeler M. D. Rusyn I. Rusyn E. V. Cox A. D. (2001). Endothelial Cells Contain a Glycine-Gated Chloride Channel. Nutr. Cancer 40 (2), 197–204. 10.1207/s15327914nc402_17 11962256

[B46] Yu S. Gao L. Song Y. Ma X. Liang S. Lan H. (2021). Glycine Ameliorates Mitochondrial Dysfunction Caused by ABT-199 in Porcine Oocytes. J. Anim. Sci. 99 (4), skab072. 10.1093/jas/skab072 33687436 PMC8075119

[B47] Zander-Fox D. Cashman K. S. Lane M. (2013). The Presence of 1 mM glycine in Vitrification Solutions Protects Oocyte Mitochondrial Homeostasis and Improves Blastocyst Development. J. Assist. Reprod. Genet. 30 (1), 107–116. 10.1007/s10815-012-9898-4 23248076 PMC3553346

[B48] Zhang J. Y. Diao Y. F. Kim H. R. Jin D. I. (2012a). Inhibition of Endoplasmic Reticulum Stress Improves Mouse Embryo Development. PLoS One 7 (7), e40433. 10.1371/journal.pone.0040433 22808162 PMC3396646

[B49] Zhang J. Y. Diao Y. F. Oqani R. K. Han R. X. Jin D. I. (2012b). Effect of Endoplasmic Reticulum Stress on Porcine Oocyte Maturation and Parthenogenetic Embryonic Development *In Vitro*1. Biol. Reprod. 86 (4), 128. 10.1095/biolreprod.111.095059 22190710

[B50] Zhang L. Wang Z. Lu T. Meng L. Luo Y. Fu X. (2020). Mitochondrial Ca2+ Overload Leads to Mitochondrial Oxidative Stress and Delayed Meiotic Resumption in Mouse Oocytes. Front. Cel Dev. Biol. 8, 580876. 10.3389/fcell.2020.580876 PMC7770107 33384990

[B51] Zhao L. Lu T. Gao L. Fu X. Zhu S. Hou Y. (2017). Enriched Endoplasmic Reticulum-Mitochondria Interactions Result in Mitochondrial Dysfunction and Apoptosis in Oocytes from Obese Mice. J. Anim. Sci Biotechnol 8, 62. 10.1186/s40104-017-0195-z 28781772 PMC5537973

[B52] Zhao X.-M. Hao H.-S. Du W.-H. Zhao S.-J. Wang H.-Y. Wang N. (2016). Melatonin Inhibits Apoptosis and Improves the Developmental Potential of Vitrified Bovine Oocytes. J. Pineal Res. 60 (2), 132–141. 10.1111/jpi.12290 26485053

