# Supplementary material for: Glycine Ameliorates Endoplasmic Reticulum Stress Induced by Thapsigargin in Porcine Oocytes
Source: Front Cell Dev Biol. 2021 Nov 30;9:733860. doi: 10.3389/fcell.2021.733860 (PMC8670231; doi:10.3389/fcell.2021.733860)
Supplement: Supplementary file 1 [file DataSheet1.pdf]

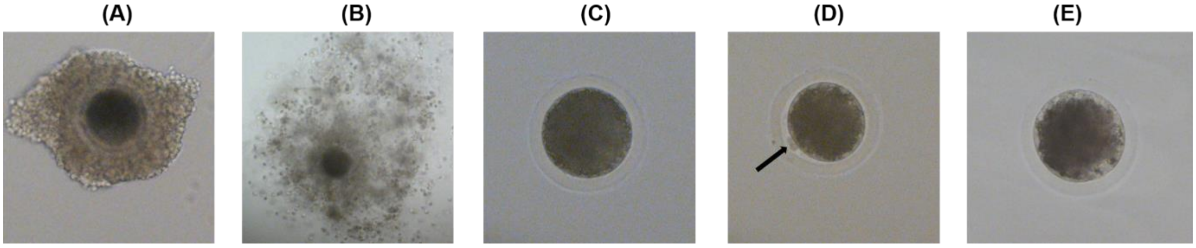

Supplementary Figure 1 *In vitro* development of porcine oocytes. The discharge of the PB1 was the marker of oocyte maturation. (A) Primordial oocyte (B) Cumulus diffusion oocyte (C) Immature oocyte (D) Mature oocyte (E) Dead oocyte

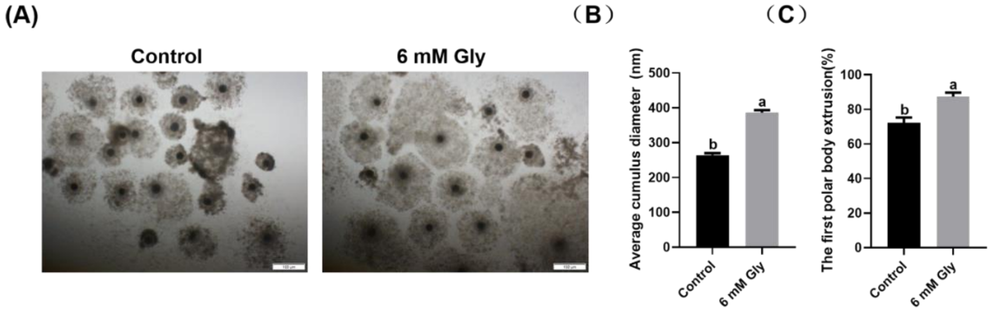

Supplementary Figure 2. Effect of Gly treatment on cumulus expansion and maturation rate during *in vitro* maturation. (A, B) Measurement of cumulus cell expansion in COCs after Gly treatment. (C) The PB1 extrusion rate. The number of oocytes examined (649) is the total of four independent assays. Bar = 200 μm. Different superscript letters denote a significant difference ($^{a,\,b}$ $P < 0.05$).

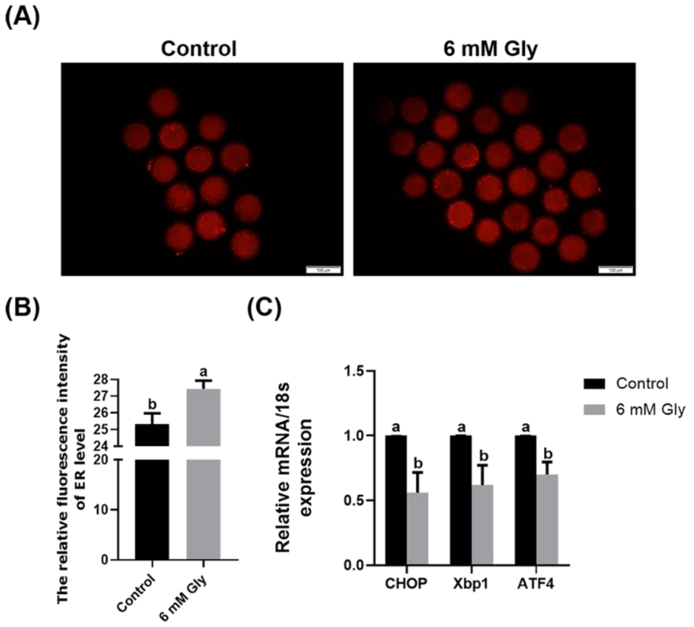

Supplementary Figure 3. Gly treatment enhances ER levels in porcine oocytes. (A, B) ER-Tracker Red (Red) signals in MII porcine oocytes. (C) The mRNA expression levels of CHOP, Xbp1 and ATF4 in mature oocytes after 6 mM Gly supplementation during *in vitro* maturation Different superscript letters denote a significant difference ($^{a,\,b}$ $P < 0.05$).

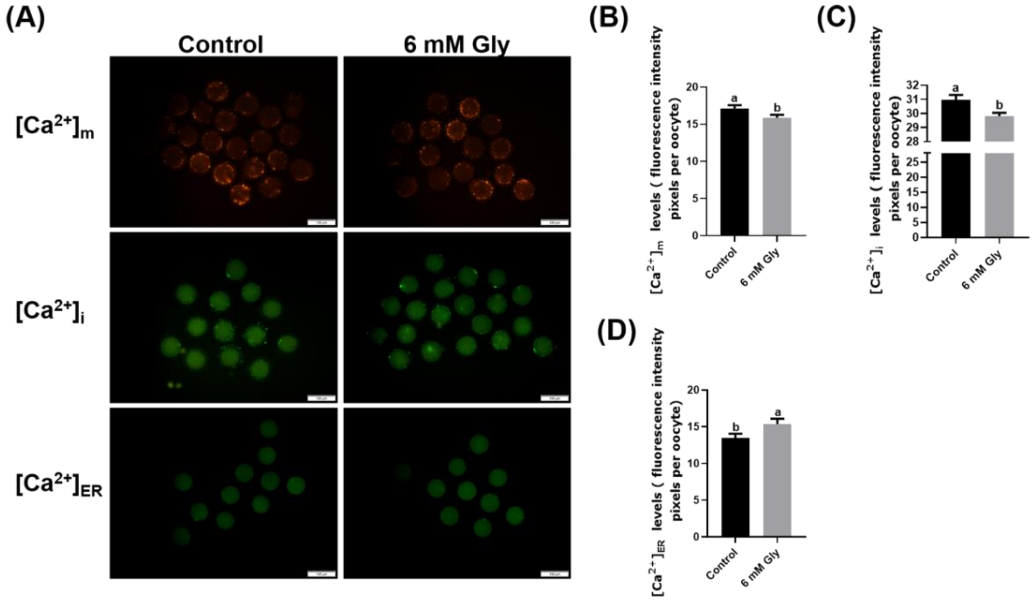

Supplementary Figure 4. Gly treatment changed $[Ca^{2+}]_i$, $[Ca^{2+}]_{ER}$, and $[Ca^{2+}]_m$ levels in porcine oocytes. (A, B, C, D) The $[Ca^{2+}]_i$ levels in MII porcine oocytes were detected by immunostaining with 5 μM Fluo-3/AM (green); the $[Ca^{2+}]_{ER}$ levels were detected by immunostaining with 10 μM Mag-Fluo-4 AM (green); the $[Ca^{2+}]_m$ levels were detected by immunostaining with 10 μM Rhod-2, AM (orange). Those were all measured by ImageJ software. ($^{a, b}$ P< 0.05).

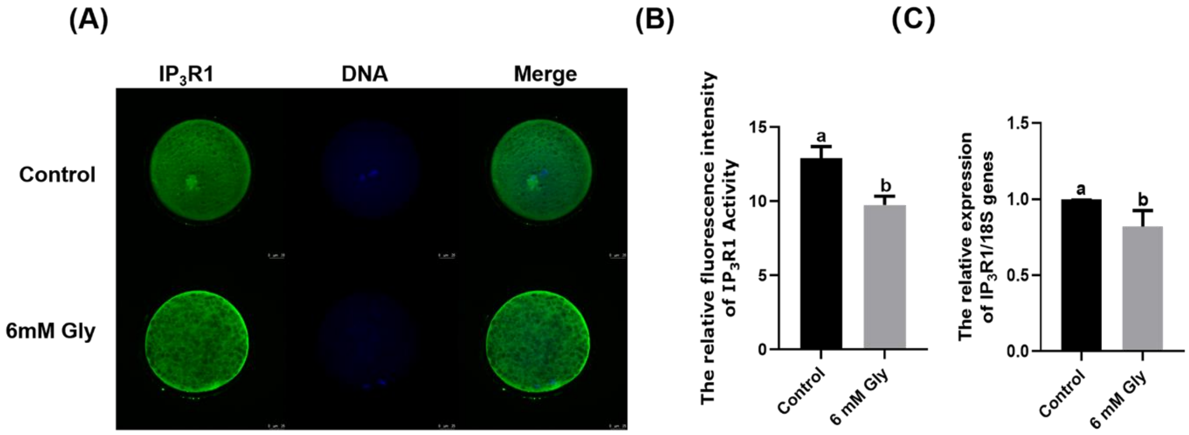

Supplementary Figure 5 Effect of Gly treatment on IP_3R1 cellular distribution in porcine oocytes *in vitro* maturation. (A, B) Gly enhances the aggregation of IP_3R1 in the cellular cortex and decreases the expression of IP_3R1. IP_3R1 (green), DNA (blue), and merged images in MII oocytes. (C) The mRNA expression levels of IP_3R1 in mature oocytes after 6 mM Gly supplementation during *in vitro* maturation Different superscript letters denote a significant difference ($^{a, b}$ $P < 0.05$).
